# Whole-brain monosynaptic outputs and presynaptic inputs of GABAergic neurons in the vestibular nuclei complex of mice

**DOI:** 10.3389/fnins.2022.982596

**Published:** 2022-08-26

**Authors:** Xun-Bei Shi, Jing Wang, Fei-Tian Li, Yi-Bo Zhang, Wei-Min Qu, Chun-Fu Dai, Zhi-Li Huang

**Affiliations:** ^1^Department of Otology and Skull Base Surgery, Eye and Ear, Nose and Throat Hospital, Fudan University, Shanghai, China; ^2^Department of Pharmacology, School of Basic Medical Sciences, Shanghai, China; ^3^State Key Laboratory of Medical Neurobiology, Ministry of Education Frontiers Center for Brain Science, Institutes of Brain Science, Fudan University, Shanghai, China; ^4^Key Laboratory of Hearing Medicine, Ministry of Health, Eye and Ear, Nose and Throat Hospital, Fudan University, Shanghai, China

**Keywords:** vestibular nuclei (VN), GABA, reciprocal neural tracing, autonomic function, sleep-wake state

## Abstract

GABAergic neurons in the vestibular nuclei (VN) participate in multiple vital vestibular sensory processing allowing for the maintenance and rehabilitation of vestibular functions. However, although the important role of GABA in the central vestibular system has been widely reported, the underlying neural circuits between VN GABAergic neurons and other brain functional regions remain elusive, which limits the further study of the underlying mechanism. Hence, it is necessary to elucidate neural connectivity based on outputs and inputs of GABAergic neurons in the VN. This study employed a modified rabies virus retrograde tracing vector and cre-dependent adeno-associated viruses (AAVs) anterograde tracing vector, combined with a transgenic VGAT-IRES-Cre mice, to map the inputs and outputs of VN GABAergic neurons in the whole brain. We found that 51 discrete brain regions received projections from VN GABAergic neurons in the whole brain, and there were 77 upstream nuclei innervating GABAergic neurons in the VN. These nuclei were mainly located in four brain regions, including the medulla, pons, midbrain, and cerebellum. Among them, VN GABAergic neurons established neural circuits with some functional nuclei in the whole brain, especially regulating balance maintenance, emotion control, pain processing, sleep and circadian rhythm regulation, and fluid homeostasis. Therefore, this study deepens a comprehensive understanding of the whole-brain neural connectivity of VN, providing the neuroanatomical information for further research on the neural mechanism of the co-morbidities with vestibular dysfunction.

## Introduction

The vestibular nuclei (VN) is a heterogeneous nucleus complex, mainly constituting the superior vestibular nucleus (SuVe), the spinal vestibular nucleus (SpVe), the medial vestibular nucleus (MVe), and the lateral vestibular nucleus (LVe) according to the cytoarchitecture, as well as other small cell clusters, like X and Y cells (Brodal and Pompeiano, 1957). VN participates in vital physiological behaviors as the second-order neurons that receive the sensory innervation from the peripheral vestibular system. Previous studies have widely proved that the involvement of the VN in various central pathways responded to diverse sensory activities, including balance maintenance, spatial orientation, and gaze stabilization. In addition, the stimulation of the VN has been also linked to the alteration of circadian rhythm and autonomic function, such as the sleep–wake cycle, emotional states (such as fear, anxiety, and depression) as well as pain conditions. Despite several studies to demonstrate the impact of the VN on sensory processes, the underlying neurotransmitters and neural circuits remain elusive.

Based on previous results of *in situ* hybridization and the reverse transcription PCR (RT-PCR), gamma-aminobutyric acid (GABA), one of the common inhibitory neurotransmitters, was expressed in all vestibular sub-nuclei and the GABA-releasing neurons accounted for 72.95% of the total neurons in the VN (Takazawa et al., 2004; Highstein and Holstein, 2006). Additionally, the effect of VN GABAergic neurons on multiple vestibular sensory processing allowed for the maintenance and rehabilitation of vestibular functions. The change of GABA in the VN in the normal aging process resulted in a decrease in visual accuracy and unsteady standing *via* the vestibular-ocular reflex and vestibulo-spinal reflex pathways (Smith, 2016). More importantly, after the damage of the peripheral vestibular receptors, the vestibular dynamic and static symptoms gradually disappeared along with the GABA distribution back to the normal level (Li et al., 1996; Gliddon et al., 2005; Bergquist et al., 2008). In addition, recent studies have also found that 5-HT, which mediated emotion regulation, also regulated the GABA release through 5-HT receptors on the VN, thereby altering the spontaneous inhibitory postsynaptic potential to affect the vestibular function (Ahn et al., 2009; Han et al., 2021). VN GABAergic neurons were also involved in the vestibular-autonomic pathway to regulate autonomic function (Holstein et al., 2016). In summary, GABA functions as a key neurotransmitter in the VN involved in multisensory and multimodal vestibular processing *via* different vestibular pathways. However, although the important role of GABA in the central vestibular system has been generally extensively studied, the lack of comprehensive neural circuits between VN GABAergic neurons and functional brain regions limits the further study of the underlying mechanism.

During the last century, although several studies have provided invaluable information in the characterization of important brain areas with monosynaptic projections with the VN using the conventional tracing approaches (such as cholera toxin subunit B, Phytohemagglutinin-L, and Fluoro-Gold), yet these studies have been not enough to determine a full understanding of cell-type-specific neurons in the VN limited by utilization of non-specific tracing vector and wild-type mice. For example, traditional tracing findings could not distinguish between glutamatergic neurons and GABAergic neurons in the VN. Therefore, to explore the underlying neural mechanism of multisensory vestibular processing, it is necessary to clarify neural connectivity based on outputs and inputs of a defined neuron type rather than of a defined anatomical region. Our previous study has elucidated the whole-brain mapping atlas of VN glutamatergic neurons and analyzed the potential neural circuit of the co-morbidities with vestibular dysfunction (Shi et al., 2021).

This study employed a modified rabies virus retrograde tracing vector and cre-dependent adeno-associated viruses (AAVs) anterograde tracing vector, in conjunction with a transgenic VGAT-IRES-Cre mice, enabling to selectively target GABAergic neurons located in the VN. We aim to map the inputs and outputs of VN GABAergic neurons in the whole brain and uncover previously unidentified neural circuits, which will further demonstrate the presence of neuroanatomical evidence for the neural connectivity between multisensory functional nuclei and VN GABAergic neurons.

## Materials and methods

### Animals

Adult male vesicular GABA transporter (VGAT)-IRES-Cre mice from C57BL/6J mice background (12–16 weeks old, 20–29 g) (Jackson Lab Stock No. 028862) and wildtype littermates (12–16 weeks old, 20–29 g) were used in this study. Constant environmental conditions (temperature of 22 ± 0.5°C, 60 ± 2% humidity) were provided. The animals were housed in individual cages under a 12 h light: 12 h dark cycle (lights on at 07:00 am; illumination intensity, ~100 lux) (Zhang et al., 2019). Food and water were available *ad libitum*. All experimental protocols were approved by the Committee on the Ethics of Animal Experiments of Fudan University (Permit Number: 20140226-024).

### Virus and surgery

AAV2/9-EF1α-DIO-ChR2-mCherry was purchased from Taiertu (Shanghai, China). The virus titer of AAV2/9-EF1α-DIO-ChR2-mCherry was 3.0^*^10^13^ vg/ml. rAAV2/9-Ef1α-DIO-RVG-WPRE-pA, rAAV2/9-Ef1α-DIO-His-EGFP-2a-TVA-WPRE-pA, and RV-ENVA-ΔG-dsRed were packaged from BrainVTA (Wuhan, China). Before injection, rAAV2/9-Ef1α-DIO-RVG-WPRE-pA and rAAV2/9-Ef1α-DIO-His-EGFP-2a-TVA-WPRE-pA were mixed with a ratio of 1:1 as the AAV helper, with the titer of 3.5^*^10^12^ vg/ml. RV-ENVA-ΔG-dsRed had a titer of about 4.0^*^10^8^ vg/ml. All viral vectors and surgical procedures were described in our previous study (Shi et al., 2021).

VGAT-Cre mice were anesthetized with chloral hydrate (360 mg/kg) and fixed in a stereotaxic apparatus. The coordinate of the VN conformed to the second edition of George Paxinos mouse brain atlas (Paxinos and Franklin, 2001). The exposure of the skin on the top of the mouse's head was ready and the glass micropipette hung over the VN (VN bregma: anterior–posterior: −6.0 mm, medial-lateral: +0.9 mm, dorsal-ventral: −3.3 mm). For anterograde tracing, AAV2/9-hEF1a-DIO-ChR2-mCherry was injected into the right VN using a micropipette pump controller at a rate of 35 μl/10 min. To prevent the virus from spreading to the surrounding area, the pipette was held in place for 10 min after the end of the injection and retract the micropipette (Guo et al., 2020). Three weeks later, all mice were perfused. Following similar surgical procedures, retrograde neural tracing was performed *via* the injection of AAV helper (rAAV2/9-Ef1α-DIO-RVG-WPRE-pA+rAAV2/9-Ef1α-DIO-His-EGFP-2a-TVA-WPRE-pA) into the right VN with a micropipette pump controller (rate of 35 μl/10 min). Three weeks later, RV-ENVA-ΔG-dsRed was injected into the same coordinate in the same way. One week after the second injection, all mice were perfused.

### Histology and immunostaining

The mice were anesthetized with 1.5% isoflurane in oxygen (flow rate of 1 L/min) with the anesthetic mask and then were perfused with 0.01 M PBS and 4% paraformaldehyde (PFA) in turn. Next, the brain tissue was quickly extracted from the skull and put into 4% PFA for post-fixation overnight at 4°C. Then, the brain tissue was incubated in the 20 and 30% sucrose successively at 4°C until completely sank. Dehydrated brains were embedded into the O.C.T. compound and retained under a frozen condition at −80°C. Tissues were cut into 30 μm serial coronal sections on the freezing cryostat (CM1950, Leica, Germany), divided into equal three groups, and stored in 0.01M PBS at 4°C.

For retrograde tracing, sections were washed with 0.01M PBS three times, mounted on the slides, and then observed under the fluorescence microscope. For anterograde tracing, sections were also washed with 0.01M PBS 3 times and incubated by the primary antibody (rabbit anti-mCherry, 1:3,000, 632496, Takara, Japan) overnight in a constant temperature (4°C) shaker incubator. Next, the sections were washed and incubated in the biotinylated goat anti-rabbit IgG (1:1,000, Invitrogen, Carlsbad, CA) in 0.3% PBST for 1 h at room temperature (RT). Sections were then washed three times and incubated in the mixture of 1 μL avidin and 1 μL biotin (avidin–biotin: 0.01M PBS = 1:1,000, Vector Laboratories, Burlingame, CA) for 1 h. After being washed, sections were counterstained with 3,3-diaminobenzidine-4 HCL (DAB) (Vector Laboratories, Burlingame, CA) solution until the appearance of yellowish-brown axonal terminals and neurons in the injection location. Finally, the stained sections were completely washed, mounted on the slides, dehydrated at RT, coverslipped, and stored at 4°C.

### Imaging and analysis

The images of whole-brain sections were detected by the 10X objective on the Olympus VS120 microscope (Tokyo, Japan). The second version of George Paxinos mouse brain atlas helps to define the border of each brain subregion. We defined the location of dsRed-labeled neurons and quantified these afferent neurons using the Image J software. The proportion of dsRed-labeled neurons was calculated by the ratio of the average number of dsRed-labeled neurons in one nucleus to the total number of dsRed-labeled neurons in one brain. Similarly, we used the Image J software to count the number of DAB-staining axonal varicosities with stained neurons excluded. The proportion of axonal varicosities neurons was calculated by the ratio of the average number of varicosities in one nucleus to the total number of varicosities in one brain. The proportion of varicosities or dsRed-labeled neurons above 3%, from 1 to 3%, and under 1% was defined as dense, moderate, and sparse connectivity, respectively. GraphPad Prism 8.0 was used to compare the number of axonal varicosities or dsRed-labeled neurons for the descriptive statistical analysis. All data were presented as the means ± SEM.

## Results

### Identification of axonal projections of GABAergic neurons in the vestibular nuclei using a specific anterograde tracing strategy

**Figure 1:**
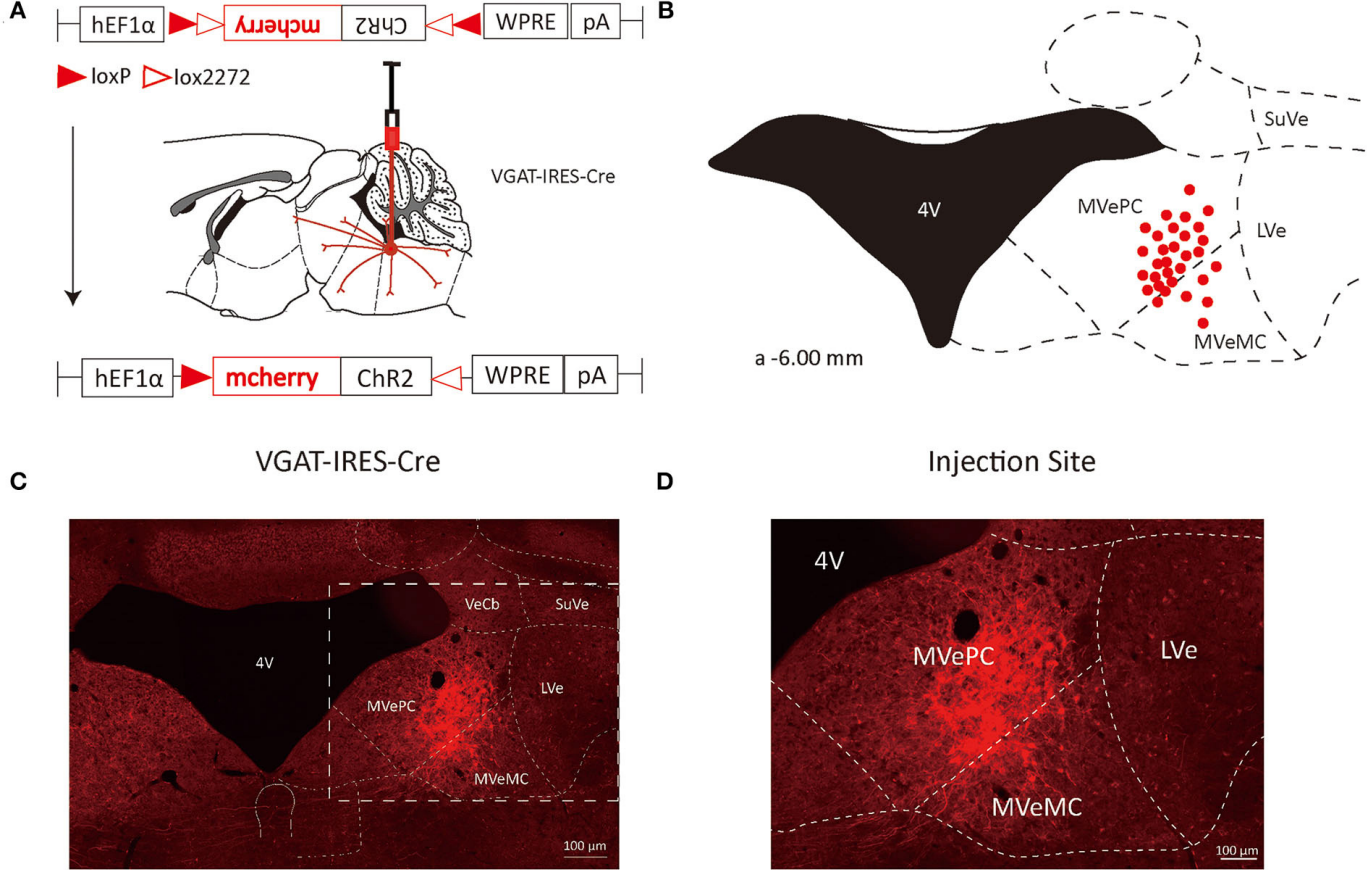
VN GABAergic neurons in the Vgat-ires-cre mice with a modified AAV-based, anterograde tracing vector. **(A)** Schematic diagram of anterograde tracing of VN GABAergic neurons using AAV vector, and the overall outputs distribution of VN GABAergic neurons (sagittal). **(B)** After injection into the VN, schematic representation of positive GABAergic neurons (red); **(C,D)** Typical figures showing the injection site of the recombinant anterograde vector for VN GABAergic neurons of VGAT-IRES-Cre mice. Scale Bar: 100 μm.

**Figure 2:**
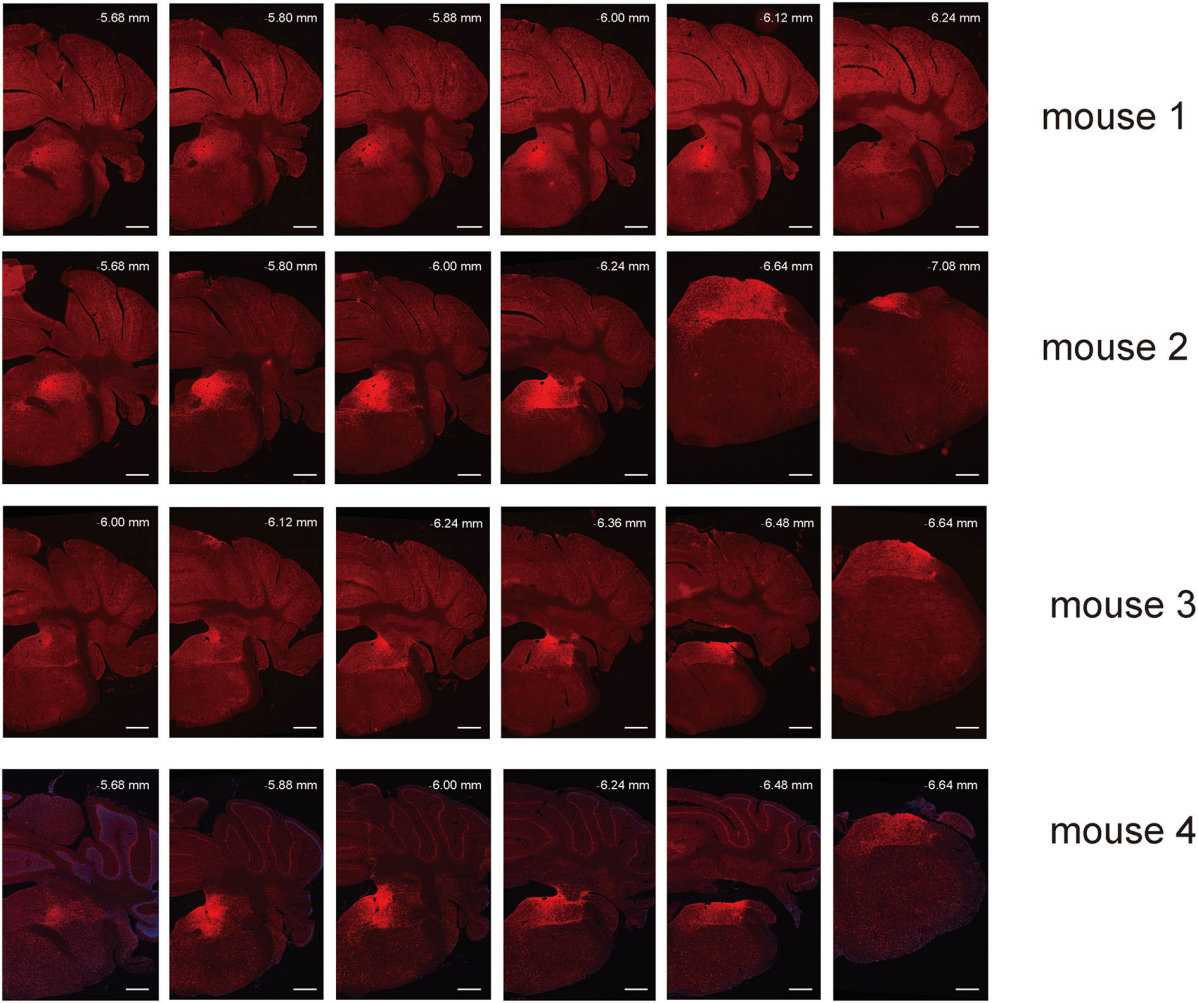
Spread of AAV anterograde tracing vector in VN GABAergic neurons in four different VGAT-IRES-Cre mice. Scale Bar: 10 μm.

To study the axonal terminal distribution of VN GABAergic neurons in the whole brain, we used recombinant adeno-associated virus for anterograde tracing in VGAT-IRES-Cre mice based on the cre/LoxP system. We injected the anterograde tracing vector (AAV2/9-hEF1a-DIO-ChR2-mCherry) into the VGAT-IRES-Cre mice, mCherry was specifically expressed in GABAergic neurons (Figures 1, 2). After 3 weeks, mice were perfused and brains were harvested. mCherry in GABAergic neurons spread along axons to axonal terminals, which could be detected under the microscope. And then, we counterstained whole-brain sections with DAB immunohistochemistry to better visualize axonal terminals distribution. Meanwhile, we injected AAV2/9-hEF1a-DIO-ChR2-mCherry into the VN of wild-type mice in the control group and found no mCherry expression in any neurons, suggesting tracing vector specifically recognized cre expression neurons.

### Outputs distribution of VN GABAergic neurons in the whole brain

**Figure 3:**
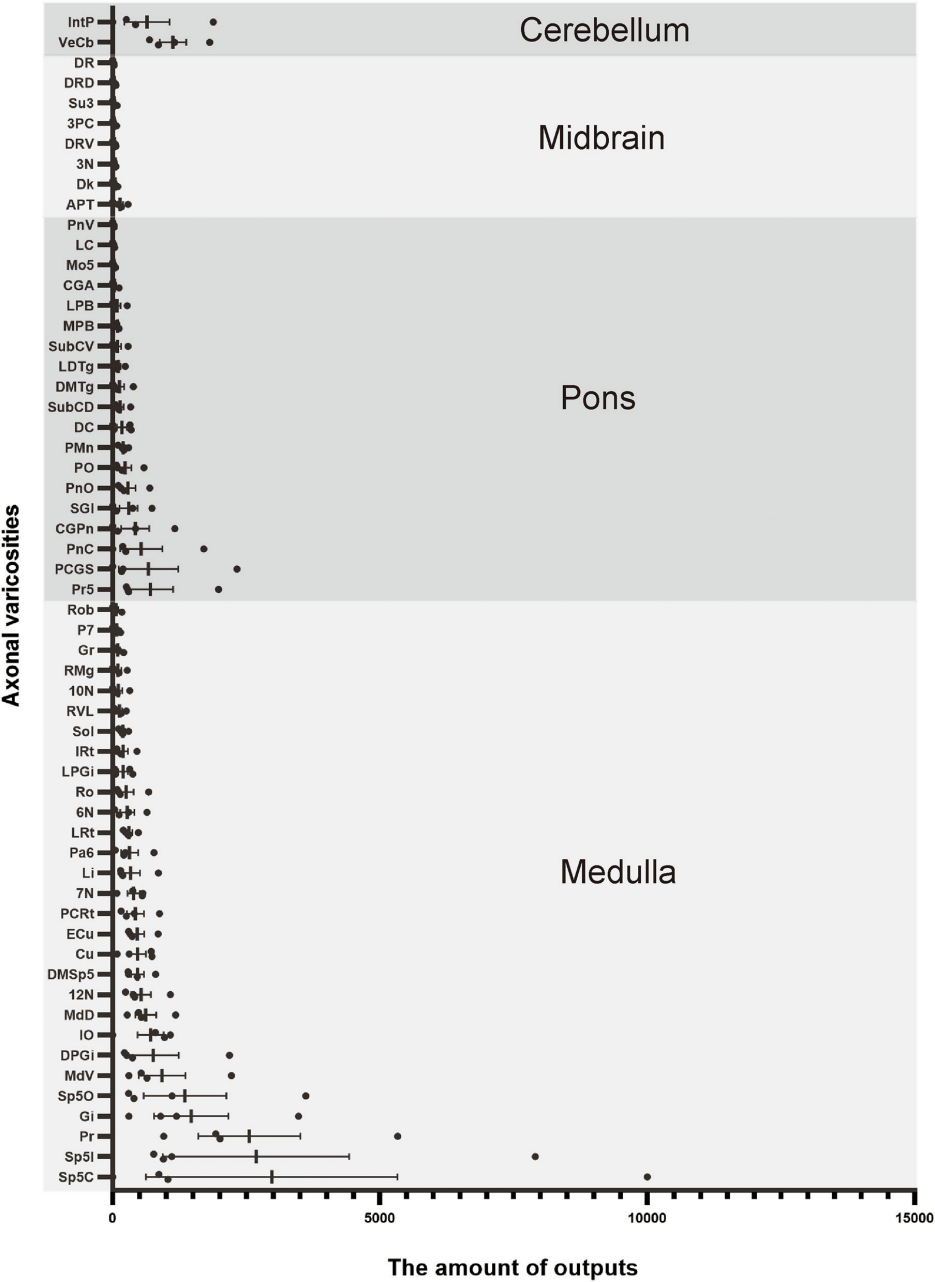
Statistical analysis of distribution of axonal terminals in the whole brain of VN GABAergic neurons in VGAT-IRES-Cre mice (*n* = 4). (Only nuclei with a ratio > 0.1% were counted). Pr, prepositus nucleus; Gi, gigantocellular reticular nucleus; DPGi, dorsal paragigantocellular nucleus; Sp5, spinal trigeminal nucleus; 7N, facial nucleus; MdV, medullary reticular nucleus, ventral part; Sol, nucleus of the solitary tract; ECu, external cuneate nucleus; PCRt, parvicellular reticular nucleus; Ro, nucleus of Roller; LPGi, lateral paragigantocellular nucleus; IRt, intermediate reticular nucleus; IO, inferior olive; P7, perifacial zone; RMg, raphe magnus nucleus; LRt, lateral reticular nucleus; MdD, medullary reticular nucleus, dorsal part; DC, dorsal cochlear nucleus; Cu, cuneate nucleus; SGI, superficial glial zone of the cochlear nuclei; 12N, hypoglossal nucleus; RVL, rostroventrolateral reticular nucleus; PnC, pontine reticular nucleus, caudal part; CGPn, central gray of the pons; PCGs, paracochlear glial substance; VeCb, vestibulocerebellar nucleus; PnO, pontine reticular nucleus, oral part; LDTg, laterodorsal tegmental nucleus; DMTg, dorsomedial tegmental area; DpMe, deep mesencephalic nucleus; Pa6, paraabducens nucleus; MnR, median raphe nucleus; DTg, dorsal tegmental nucleus; KF, Ko¨lliker-Fuse nucleus; PB, parabrachial nucleus; CGA, central gray, alpha part; CPO, caudal periolivary nucleus; Pr5, principal sensory trigeminal nucleus; LC, locus coeruleus; SPO, superior paraolivary nucleus; 6N, abducens nucleus; RtTg, reticulotegmental nucleus of the pons; VLL, ventral nucleus of the lateral lemniscus; DPO, dorsal periolivary region; APT, anterior pretectal nucleus; 3N, oculomotor nucleus; Su3, supraoculomotor periaqueductal gray; InC, interstitial nucleus of Cajal; Dk, nucleus of Darkschewitsch; RC, raphe cap; DRN, dorsal raphe nucleus; PPTg, pedunculopontine tegmental nucleus; PAG, periaqueductal gray; 3PC, oculomotor nucleus, parvicellular part; ZI, zona incerta; Lat, lateral (dentate) cerebellar nucleus; Int, interposed cerebellar nucleus; Med, medial (fastigial) cerebellar nucleus.

**Figure 4:**
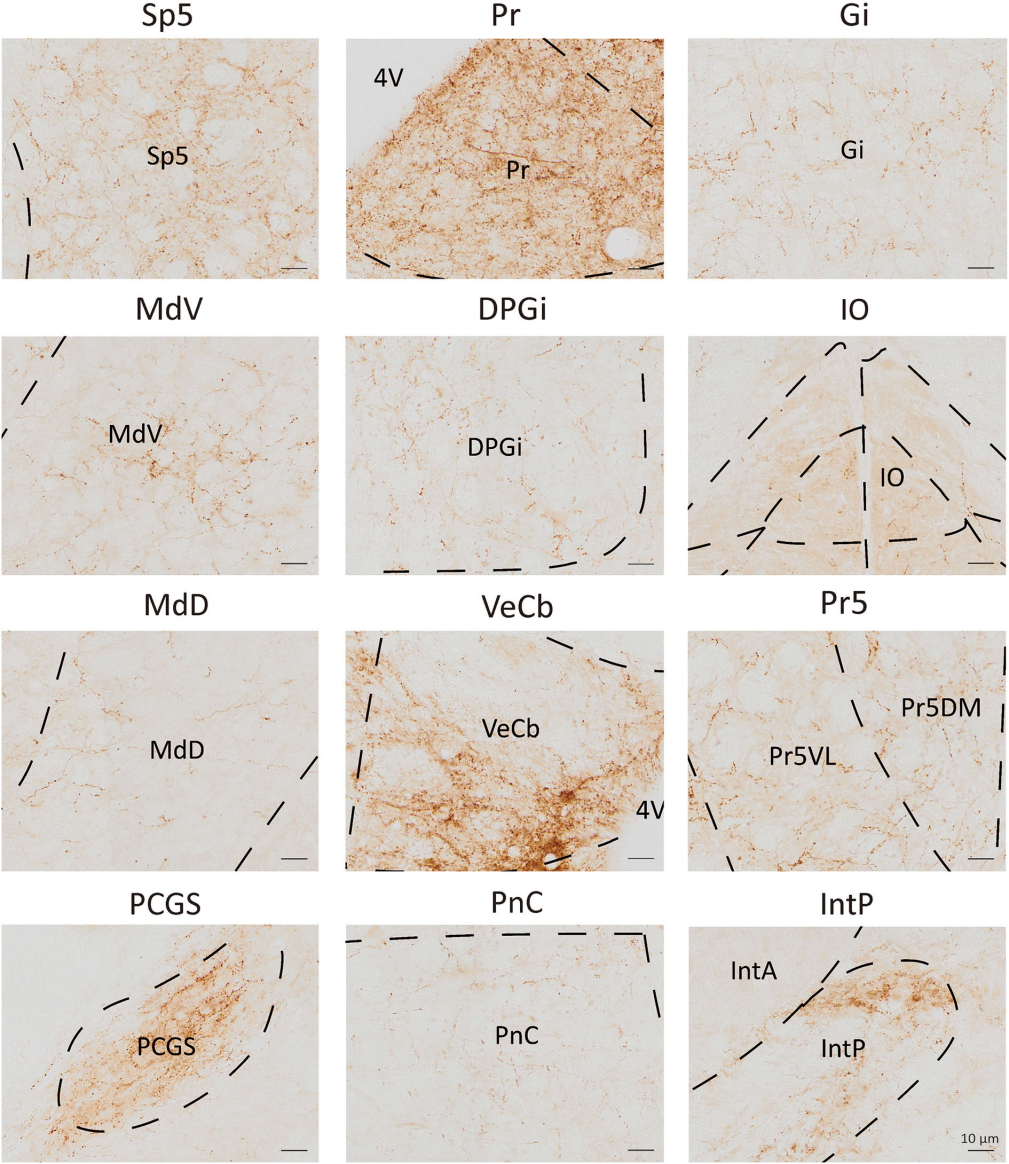
Nuclei densely projected from VN GABAergic neurons. Scale bar: 10 μm. Sp5, spinal trigeminal nucleus; Pr, prepositus nucleus; Gi, gigantocellular reticular nucleus; MdV, medullary reticular nucleus, ventral part; DPGi, dorsal paragigantocellular nucleus; IO, inferior olive; MdD, medullary reticular nucleus, dorsal part; VeCb, vestibulocerebellar nucleus; Pr5, principal sensory trigeminal nucleus; PCGs, paracochlear glial substance; PnC, pontine reticular nucleus, caudal part; IntP, interposed cerebellar nucleus, posterior part.

**Figure 5:**
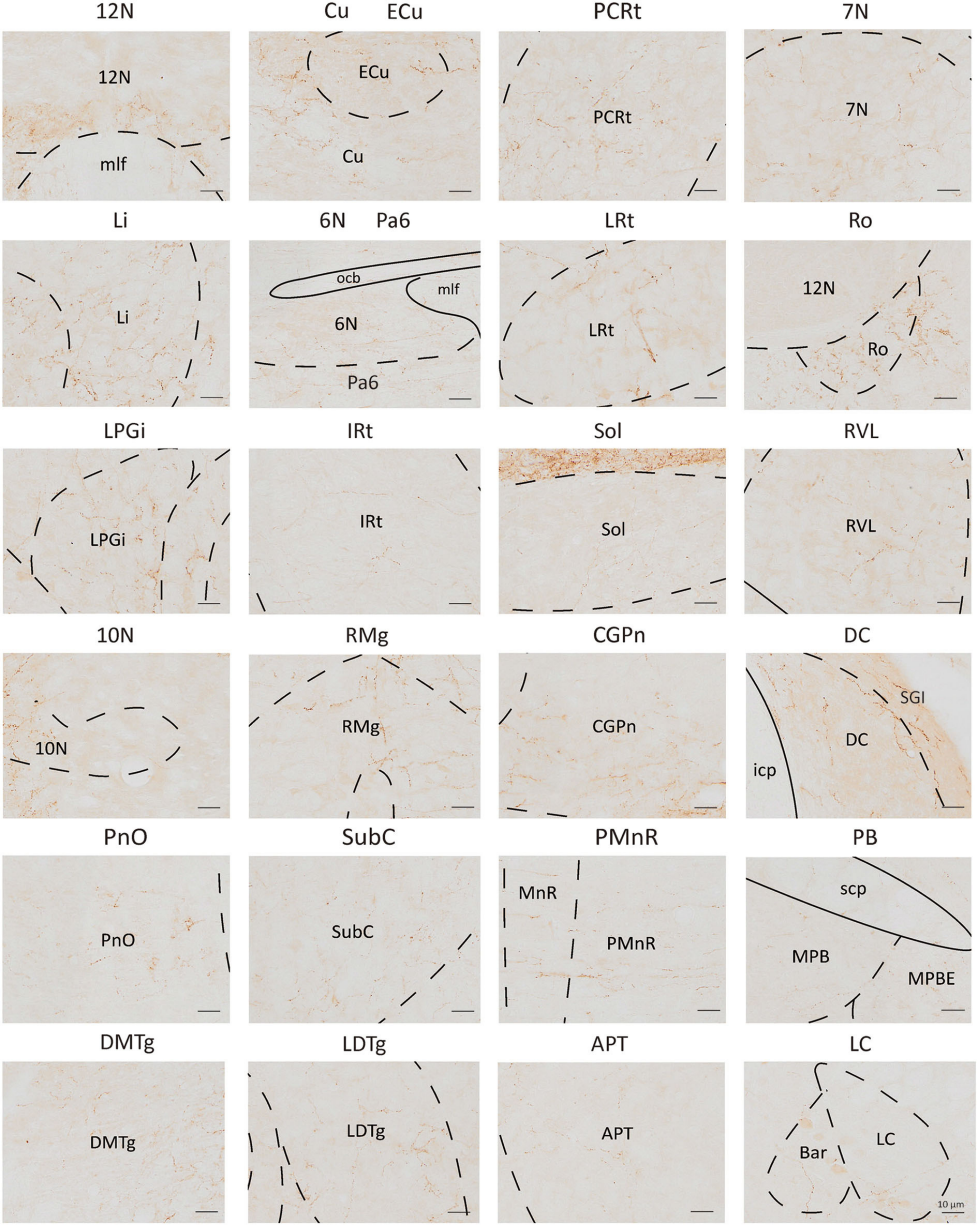
Schematic images showing nuclei received moderate to sparse outputs from VN GABAergic neurons. Scale bar: 10 μm. 12N, hypoglossal nucleus; Cu, cuneate nucleus; ECu, external cuneate nucleus; PCRt, parvicellular reticular nucleus; 7N, facial nucleus; Li, linear nucleus of the medulla; 6N, abducens nucleus; Pa6, paraabducens nucleus; LRt, lateral reticular nucleus; Ro, nucleus of Roller; LPGi, lateral paragigantocellular nucleus; IRt, intermediate reticular nucleus; Sol, nucleus of the solitary tract; RVL, rostroventrolateral reticular nucleus; 10N, dorsal motor nucleus of vagus; RMg, raphe magnus nucleus; CGPn, central gray of the pons; DC, dorsal cochlear nucleus; PnO, pontine reticular nucleus, oral part; SubC, subcoeruleus nucleus; PMnR, paramedian raphe nucleus; PB, parabrachial nucleus; DMTg, dorsomedial tegmental area; LDTg, laterodorsal tegmental nucleus; APT, anterior pretectal nucleus; LC, locus coeruleus.

To analyze the majority distribution of anterograde projection of VN GABAergic neurons, the anatomical classification of mCherry-labeled neurons was determined by the second edition of George Paxinos mouse brain atlas (Paxinos and Franklin, 2001), resulting in the detection of labeled neurons in 51 discrete brain regions. The positive axonal terminals were mainly located in the ipsilateral brain area, which occupied the majority of four regions, namely the medulla, pons, midbrain, and cerebellum (68.33, 21.76, 1.45, 8.45%, respectively) (Figure 3). To quantify the whole-brain mapping atlas, we manually calculated labeled neurons in regions from high-density labeling to sparse labeling. Sp5, Pr, Gi, MdV, DPGi, IO, MdD, VeCb, Pr5, PCGS, PnC, and IntP received the densest projections from VN GABAergic neurons (Figures 3, 4). VN GABAergic neurons sent a moderate number of outputs to 12N, Cu, ECu, PCRt, 7N, Li, 6N, Pa6, LRt, Ro, LPGi, IRt, Sol, RVL, 10N, RMg, CGPn, DC, PnO, SubC, PMnR, PB, DMTg, LDTg, APT, and LC (Figures 3, 5).

### Visualization of long-range inputs to GABAergic neurons in the vestibular nuclei using rabies virus and cre/LoxP recombination system

**Figure 6:**
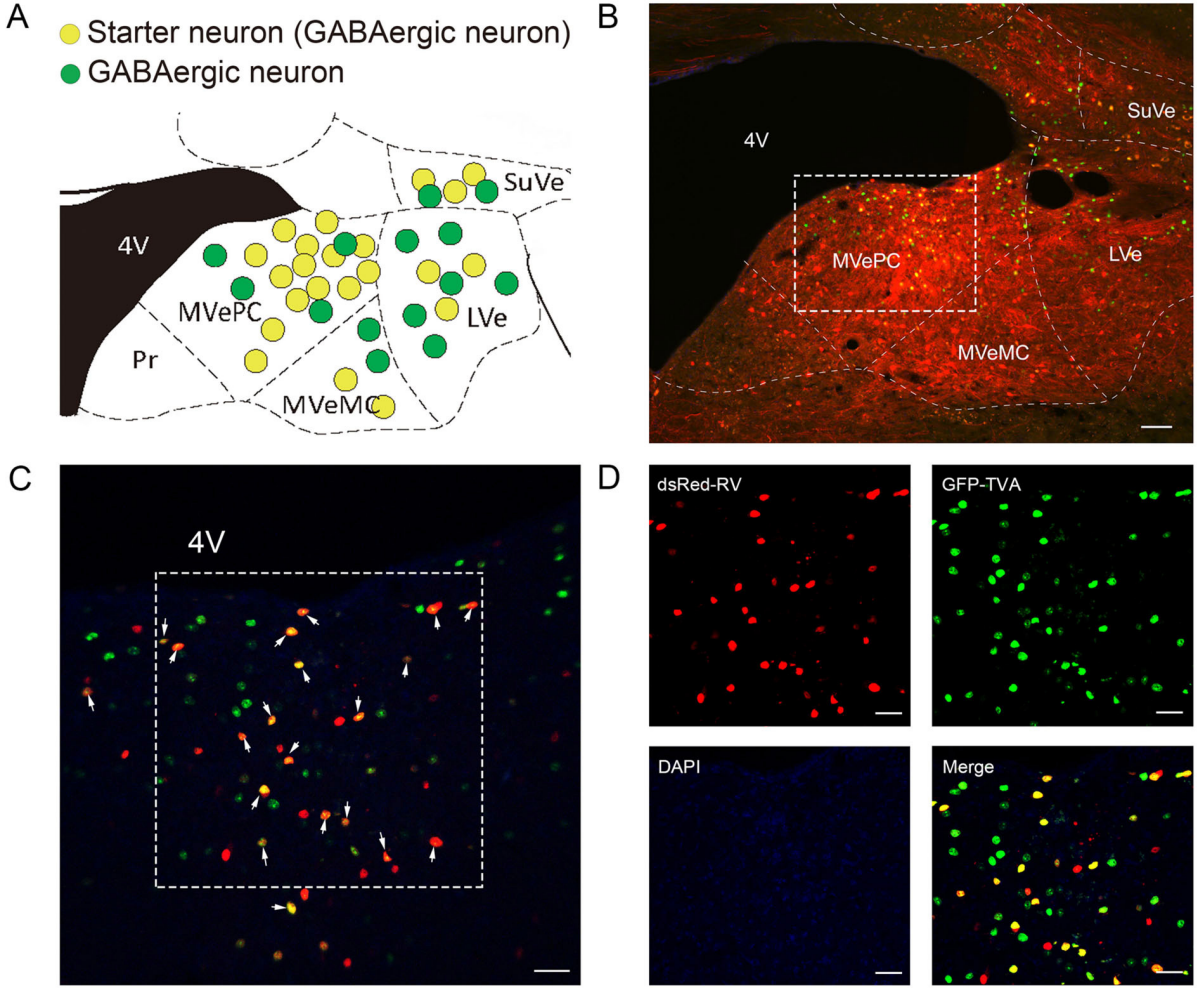
VN GABAergic neurons in the VGAT-IRES-Cre mice with a modified RV in combination with AAV helper as the retrograde tracing vector **(A)** Coronal schematic diagram of retrograde tracing of VN GABAergic neurons (starter neurons, yellow; AAV helper labeled neurons, green) (Bregma:- 6.00 mm). **(B)** The injection site of Vgat-ires-cre mice. **(C,D)** Typical figures showing the injection site showing AAV helper labeled neurons (green), starting neurons (yellow), and afferent neurons (red) under the confocal microscope; scale bar: 50 μm.

**Figure 7:**
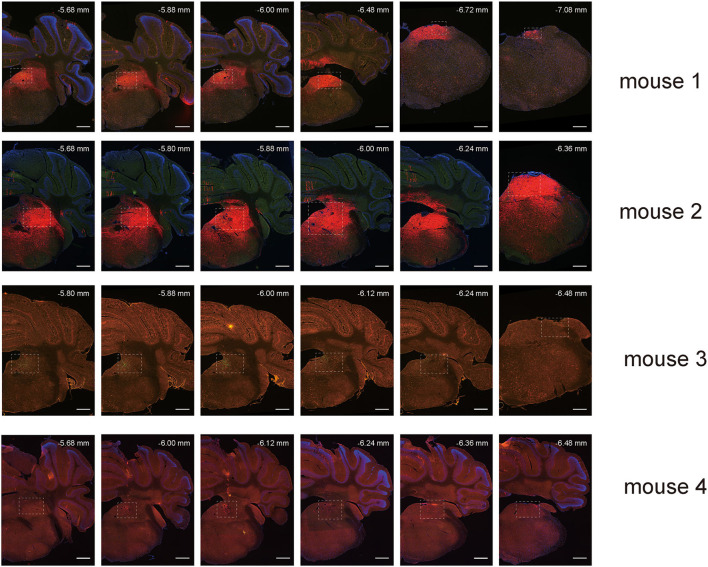
Spread of retrograde tracing vector in VN GABAergic neurons in four VGAT-IRES-Cre mice (starter neurons, orange; only AAV helper-labeled neurons, green; afferent neurons, red); scale bar: 10μm.

To map the whole-brain afferents to VN GABAergic neurons, we applied modified RV in combination with AAV helper targeting VN GABAergic neurons based on a transgenic mouse line (VGAT-IRES-Cre mice). To label the GABAergic neruons in the VN, the first injection was followed by a delivery of an AAV helper, which identified and achieved TVA receptor expression to the GABAergic neurons in the VN. Three weeks later, VGAT-IRES-Cre mice were injected with RV-ENVA-ΔG-dsRed, which was limited to infect the EGFP-labeled neurons as ENVA (avian virus envelope protein) in the RV-only recognized neurons expressing the TVA receptor. Therefore, RV could specifically identify the VN GABAergic neurons as starter cells and spread presynaptic neurons retrogradely. One week later, the brain tissue was removed and the whole-brain slices were observed under a fluorescence microscope with a 10X objective. Starter neurons were labeled with the co-expression of EGFP (green fluorescence) and dsRed (red fluorescence), whereas neurons marked with only red fluorescence in the bilateral VN represented innervating neurons that could send terminals to starter neurons (Figures 6, 7). Those neurons with EGFP expression were only infected with AAV helper but did not have the retrograde transsynaptic capability.

### Monosynaptic input patterns of VN GABAergic neurons in the whole brain

To explore the distribution of monosynaptic afferents to VN GABAergic neurons in the whole brain, we observed serial coronal brain sections of four VGAT-IRES-Cre mice. There were 77 upstream nuclei innervating GABAergic neurons in the VN. Results showed that dsRed-positive neurons were also concentrated in the ipsilateral brain region, with a few scattered in the contralateral region. DsRed-labeled neurons mainly lied in four brain regions, including the medulla, pons, midbrain, and cerebellum. In addition, some innervating nuclei in the superior colliculus, thalamus, hypothalamus, and cortex are also projected to VN GABAergic neurons.

**Figure 8:**
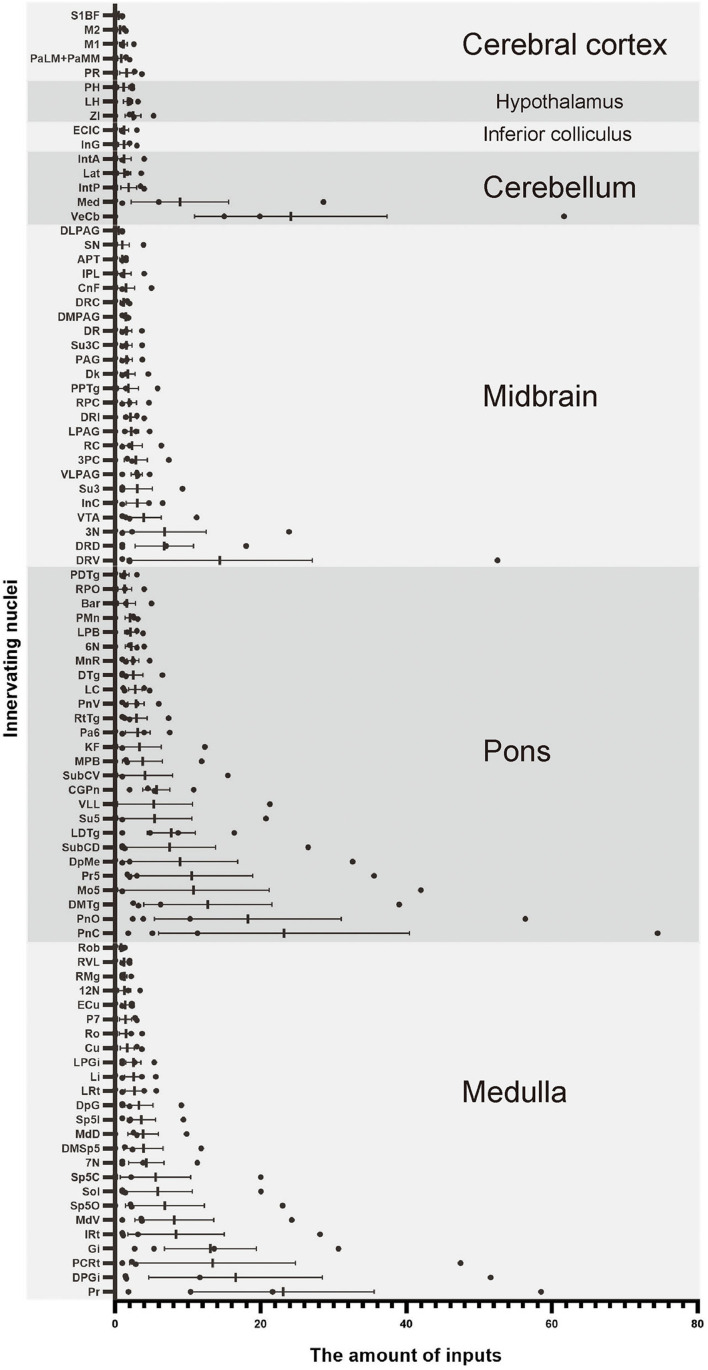
Statistical analysis of whole-brain afferent nuclei of VN GABAergic neurons in VGAT-IRES-Cre mice (*n* = 4) (Only nuclei with a ratio > 0.1% were counted). Pr, prepositus nucleus; DPGi, dorsal paragigantocellular nucleus; PCRt, parvicellular reticular nucleus; Gi, gigantocellular reticular nucleus; IRt, intermediate reticular nucleus; MdV, medullary reticular nucleus, ventral part; Sp5, spinal trigeminal nucleus; Sol, nucleus of the solitary tract; 7N, facial nucleus; MdD, medullary reticular nucleus, dorsal part; DpG, deep gray layer of the superior colliculus; LRt, lateral reticular nucleus; Li, linear nucleus of the medulla; LPGi, lateral paragigantocellular nucleus; Cu, cuneate nucleus; Ro, nucleus of Roller; P7, perifacial zone; ECu, external cuneate nucleus; 12N, hypoglossal nucleus; RMg, raphe magnus nucleus; RVL, rostroventrolateral reticular nucleus; Rob, raphe obscurus nucleus; PnC, pontine reticular nucleus, caudal part; PnO, pontine reticular nucleus, oral part; DMTg, dorsomedial tegmental area; SubC, subcoeruleus nucleus; Mo5, motor trigeminal nucleus; Pr5, principal sensory trigeminal nucleus; DpMe, deep mesencephalic nucleus; Su5, supratrigeminal nucleus; LDTg, laterodorsal tegmental nucleus; VLL, ventral nucleus of the lateral lemniscus; PB, parabrachial nucleus; CGPn, central gray of the pons; KF, Ko¨lliker-Fuse nucleus; Pa6, paraabducens nucleus; RtTg, reticulotegmental nucleus of the pons; PnV, pontine reticular nucleus, ventral part; LC, locus coeruleus; DTg, dorsal tegmental nucleus; MnR, median raphe nucleus; 6N, abducens nucleus; Bar, Barrington's nucleus; PMn, paramedian reticular nucleus; RPO, rostral periolivary region; PDTg, posterodorsal tegmental nucleus; 3N, oculomotor nucleus; DR, dorsal raphe nucleus; VTA, ventral tegmental area; InC, interstitial nucleus of Cajal; Su3, supraoculomotor periaqueductal gray; PAG, periaqueductal gray; 3PC, oculomotor nucleus, parvicellular part; RC, raphe cap; PPTg, pedunculopontine tegmental nucleus; RPC, red nucleus, parvicellular part; CnF, cuneiform nucleus; Dk, nucleus of Darkschewitsch; IPL, interpeduncular nucleus, lateral subnucleus; Su3C, supraoculomotor cap; SN, substantia nigra; APT, anterior pretectal nucleus; VeCb, vestibulocerebellar nucleus; Med, medial (fastigial) cerebellar nucleus; IntP, interposed cerebellar nucleus, posterior part; Lat, lateral (dentate) cerebellar nucleus; IntA, interposed cerebellar nucleus, anterior part; InG, intermediate gray layer of the superior colliculus; ECIC, external cortex of the inferior colliculus; ZI, zona incerta; LH, lateral hypothalamic area; PH, posterior hypothalamic area; PR, prerubral field; PaLM/PaMM, paravaentricular hpothalamic nucleus; M1, primary motor cortex; M2, secondary motor cortex; S1BF, primary somatosensory cortex, barrel field.

**Figure 9:**
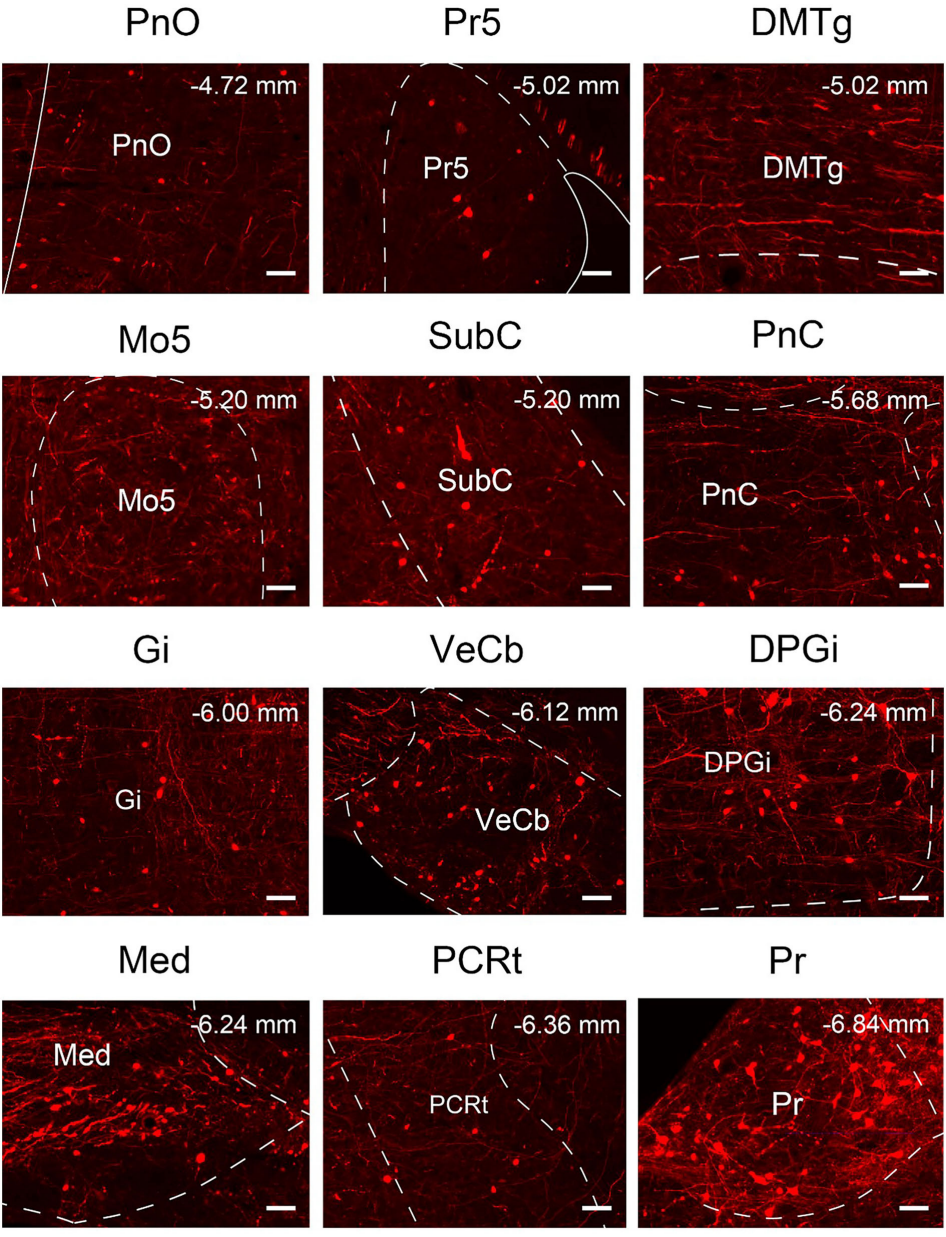
Nuclei densely projected to VN GABAergic neurons. Scale bar: 50 μm. PnO, pontine reticular nucleus, oral part; Pr5, principal sensory trigeminal nucleus; DMTg, dorsomedial tegmental area; Mo5, motor trigeminal nucleus; SubC, subcoeruleus nucleus; PnC, pontine reticular nucleus, caudal part; Gi, gigantocellular reticular nucleus; VeCb, vestibulocerebellar nucleus; DPGi, dorsal paragigantocellular nucleus; Med, medial (fastigial) cerebellar nucleus; PCRt, parvicellular reticular nucleus; Pr, prepositus nucleus.

To quantify the distribution of the innervating nuclei, we calculated the ratio of the number of dsRed-positive neurons in a single nucleus to that of all dsRed-labeled neurons in the whole brain. The tracing results revealed that VN GABAergic neurons received dense afferents from nuclei in the medulla and pons (>0.03 as a proportion of the total number of input neurons): Pr, DPGi, PCRt, Gi, PnC, PnO, Mo5, DMTg, SubC, and VeCb (Figures 8, 9). The following nuclei could also project to VN GABAergic neurons (>0.01 of total input neurons): IRt, MdV, Sp5, Sol, 7N, MdD, DpG, LRt, Li, LPGi, Su5, LDTg, VLL, PB, CGPn, KF, Pa6, RtTg, PnV, LC, DTg, MnR, 6N, 3N, DR, VTA, InC, Su3, PAG, 3PC, RC, PPTg, IntP, and ZI (Figure 8).

### Reciprocal neural connectivity of VN GABAergic neurons in the whole brain

**Figure 10:**
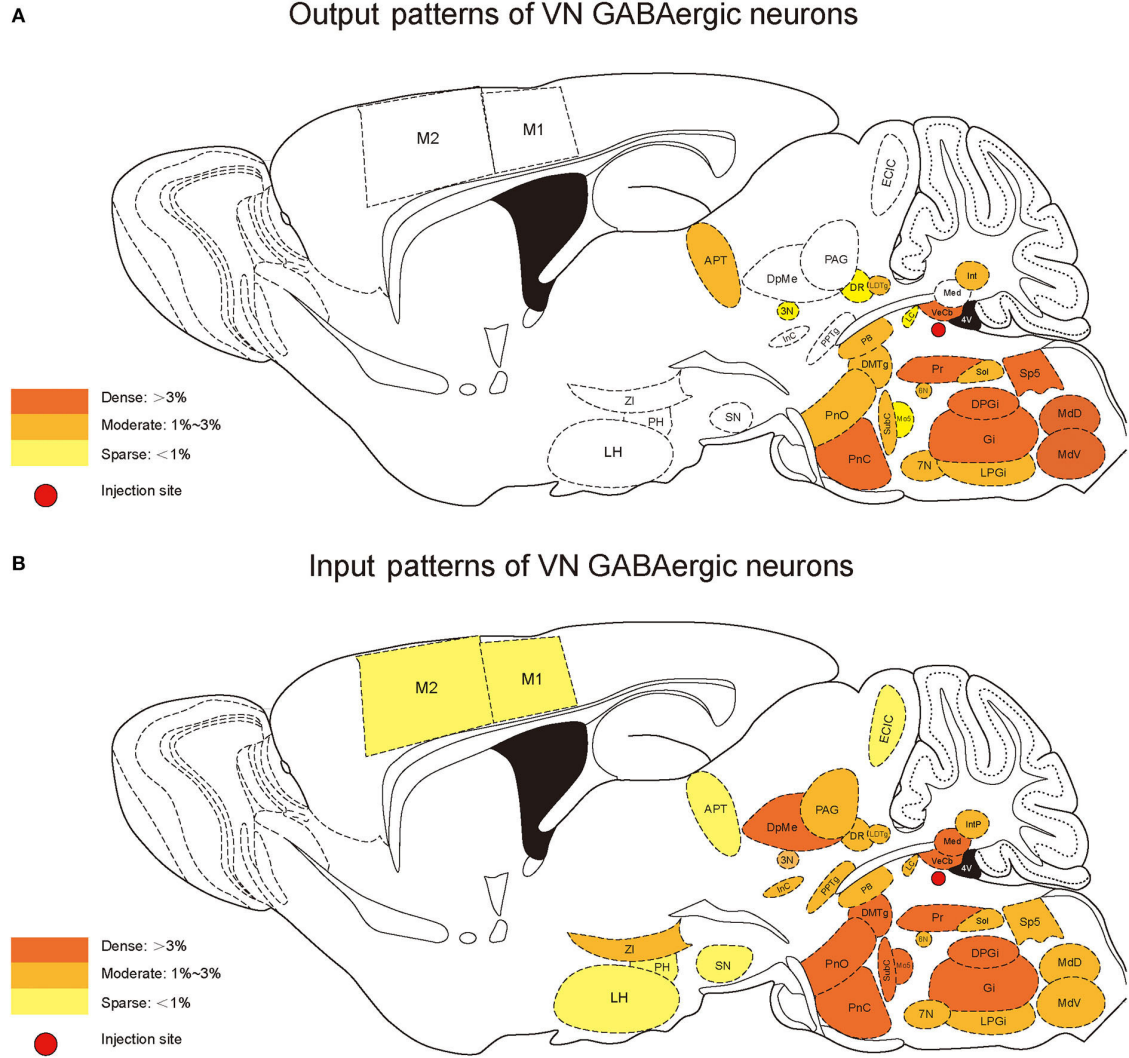
Schematic diagram of the afferent and efferent distribution of VN GABAergic neurons in mice.

According to the distribution analysis of dsRed-positive axon terminals and innervating neurons of VGAT-IRES-Cre mice, we compared the number and types of nuclei that built reciprocal neural connectivity with VN GABAergic neurons (Supplementary Table 1, Figure 10). This study showed that VN GABAergic neurons established interactive connections with 44 nuclei (mainly located in the brainstem), including Pr, DPGi, PCRt, Gi, IRt, MdV, Sp5, Sol, 7N, MdD, LRt, Li, LPGi, Cu, Ro, P7, ECu, 12N, RMg, RVL, Rob, PnC, PnO, DMTg, SubC, Mo5, Pr5, LDTg, PB, CGPn, Pa6, PnV, LC, 6N, PMnR, RPO, 3N, DR, Su3, 3PC, Dk, APT, VeCb, and IntP. Among them, Pr, DPGi, Gi, PnC, Pr5, and VeCb not only received dense afferents from VN GABAergic neurons but also innervated largely VN GABAergic neurons. Several nuclei had dense to moderate bidirectional connections to VN GABAergic neurons, namely PCRt, IRt, MdV, Sp5, Sol, 7N, MdD, LRt, Li, LPGi, PnO, DMTg, SubC, LDTg, PB, CGPn, Pa6, 6N, and IntP. In addition, VN GABAergic neurons received much more inputs from Mo5 but sent less outputs to it. On the other hand, some nuclei only link unidirectionally with VN GABAergic neurons. VN GABAergic neurons projected to IO, 10N, Gr, PCGS, SGI, DC, CPO, CGA, MVPO (medioventral periolivary nucleus), and LVPO (lateroventral periolivary nucleus), while DpG, DpMe, Su5, VLL, KF, RtTg, DTg, MnR, Bar, PDTg, VTA, InC, PAG, RC, PPTg, RPC, CnF, IPL, Su3C, SN, InG, Med, Lat, IntA, ECIC, ZI, LH, PH, PR, PaLM/PaMM, and cerebral cortex M1, M2, and S1BF could only send axonal terminals to VN GABAergic neurons (Figures 10A,B).

## Discussion

**Figure 11:**
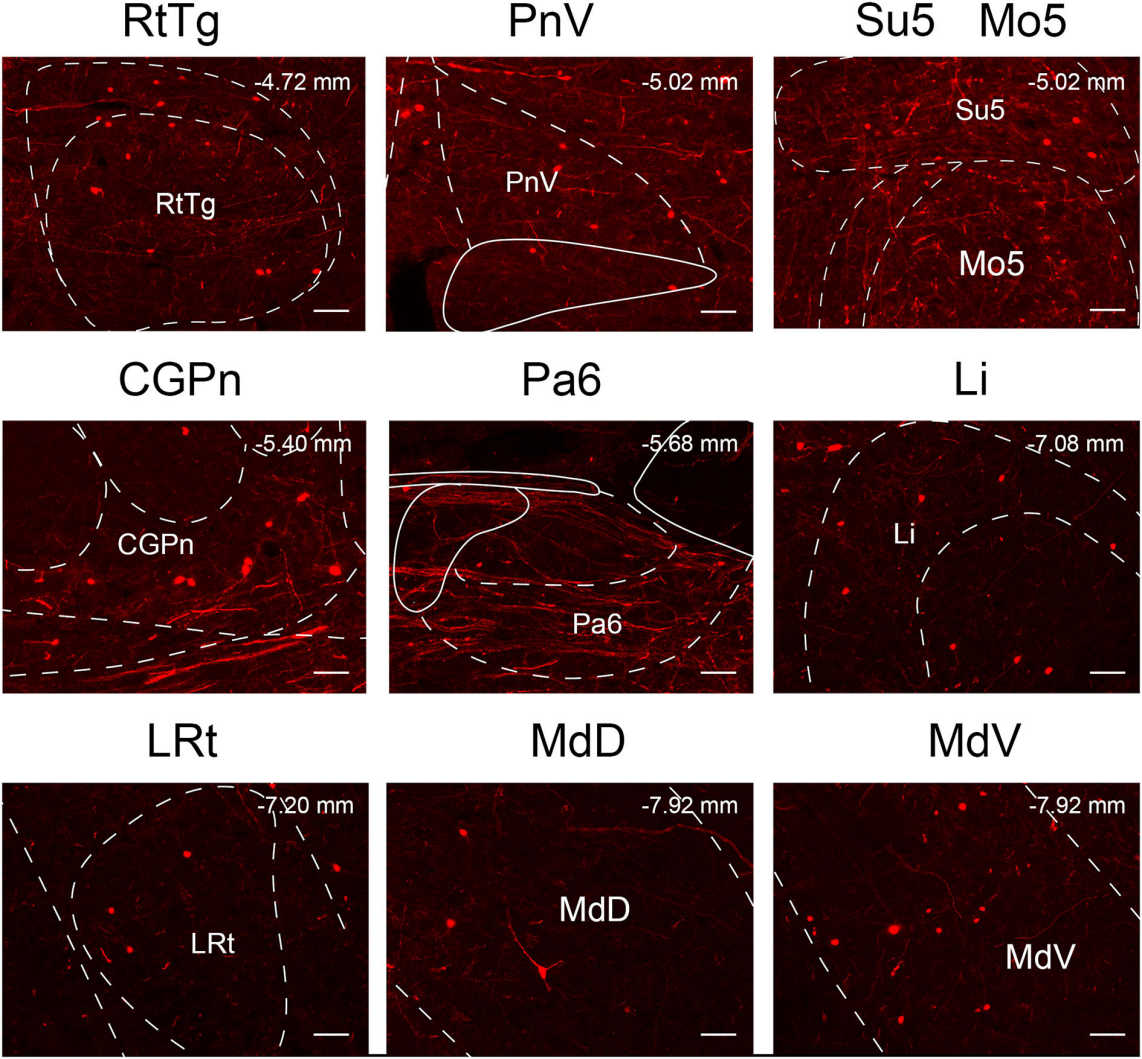
The novel nuclei sent monosynaptic inputs to VN GABAergic neurons (>0.6% of total input neurons); scale bar: 50 μm. RtTg, reticulotegmental nucleus of the pons; PnV, pontine reticular nucleus, ventral part; Su5, supratrigeminal nucleus; Mo5, motor trigeminal nucleus; CGPn, central gray of the pons; Pa6, paraabducens nucleus; Li, linear nucleus of the medulla; LRt, lateral reticular nucleus; MdD, medullary reticular nucleus, dorsal part; MdV, medullary reticular nucleus, ventral part.

**Figure 12:**
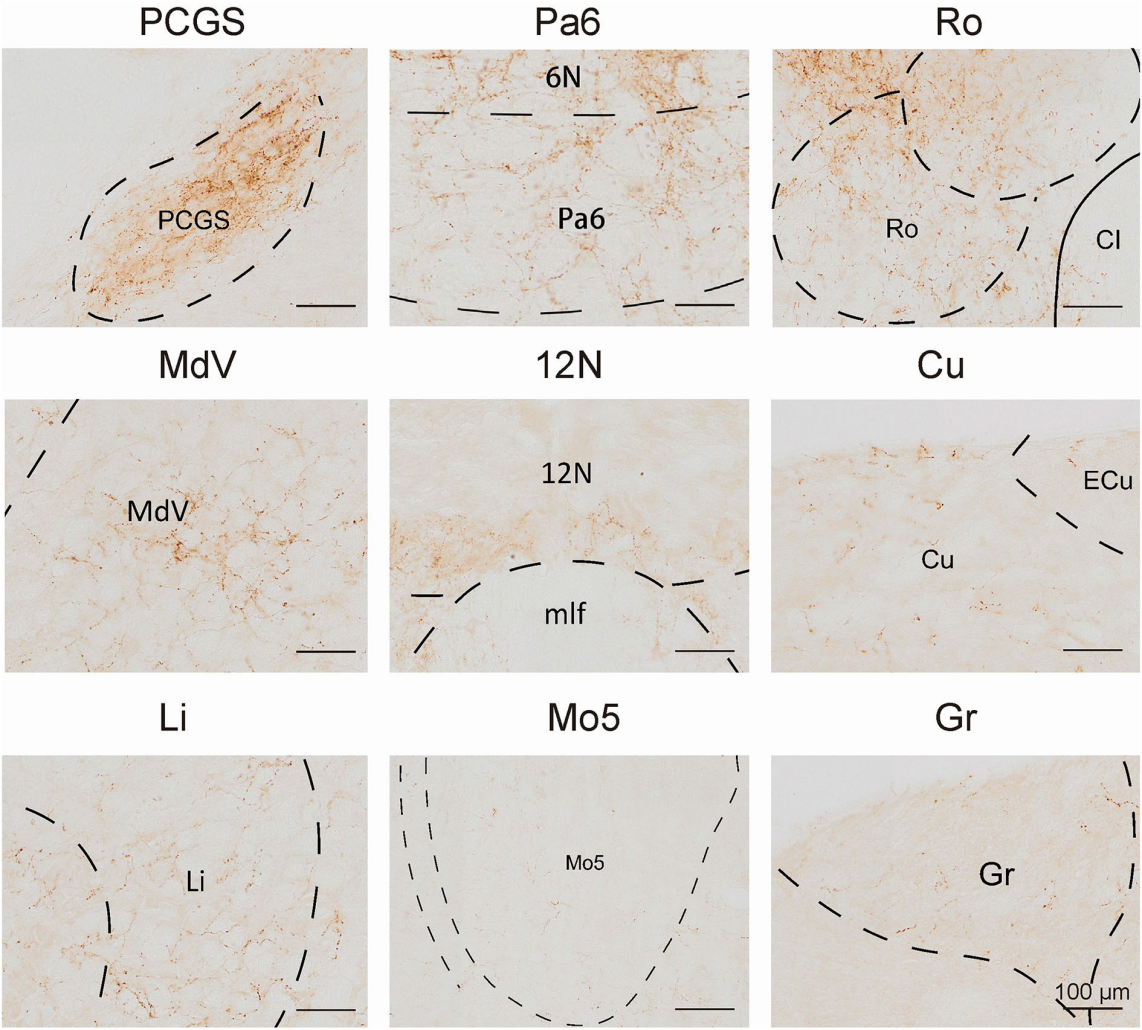
The novel nuclei received projections from VN GABAergic neurons. Scale bar: 100 μm. PCGS, paracochlear glial substance; Pa6, paraabducens nucleus; Ro, nucleus of Roller; MdV, medullary reticular nucleus, ventral part; 12N, hypoglossal nucleus; Cu, cuneate nucleus; Li, linear nucleus of the medulla; Mo5, motor trigeminal nucleus; Gr, gracile nucleus.

**Figure 13:**
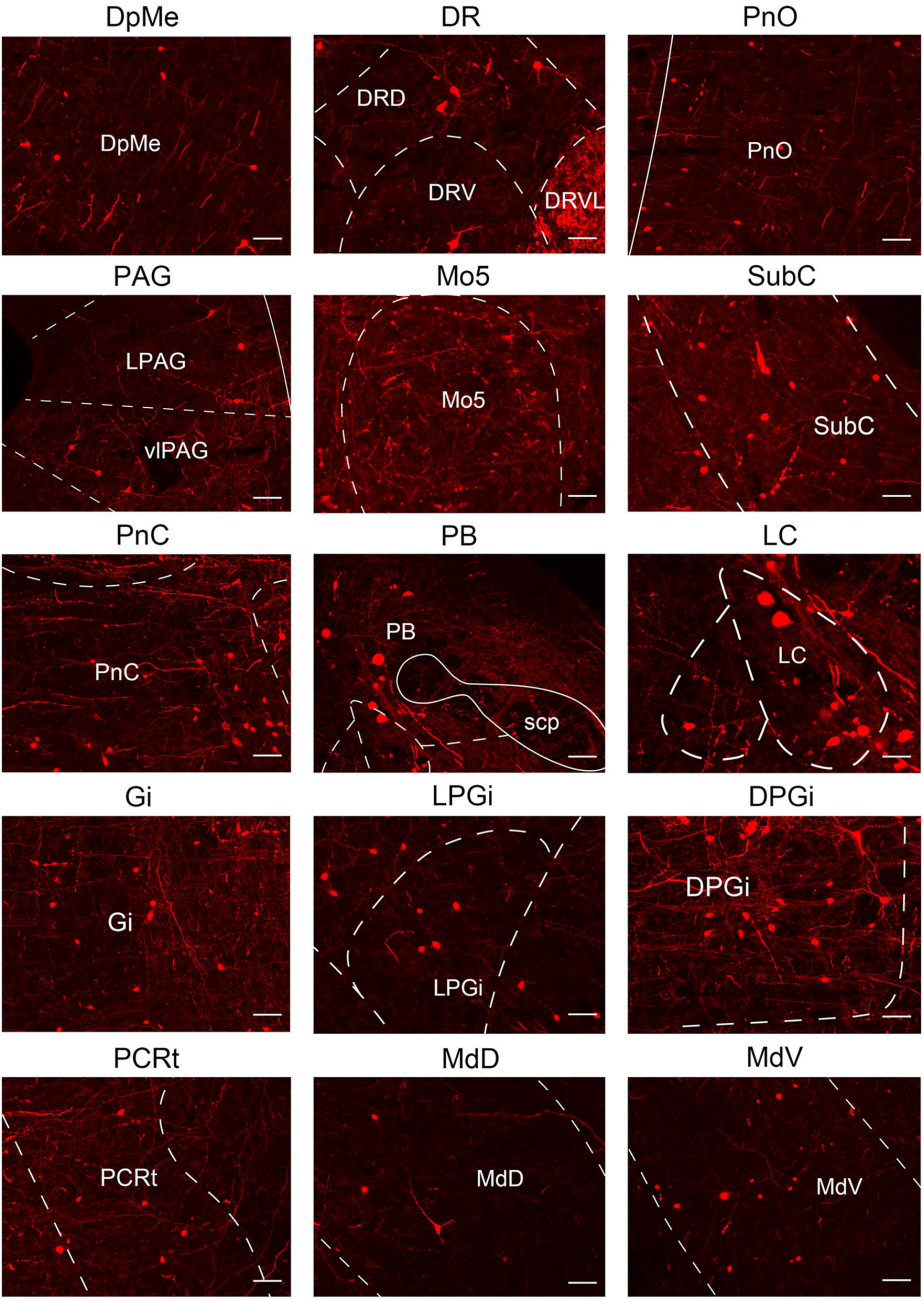
Multisensory functional nuclei that innervated VN GABAergic neurons. VN GABAergic neurons received inputs from nuclei that regulated the sleep-wake cycle (e.g., DpMe, PnO, Mo5, SubC, PnC, Gi, LPGi, DPGi, PCRt, MdD, and MdV), involved in pain regulation (PAG and PB) and emotion regulation (e.g., LC, PAG, and DR); scale bar: 50 μm.

**Figure 14:**
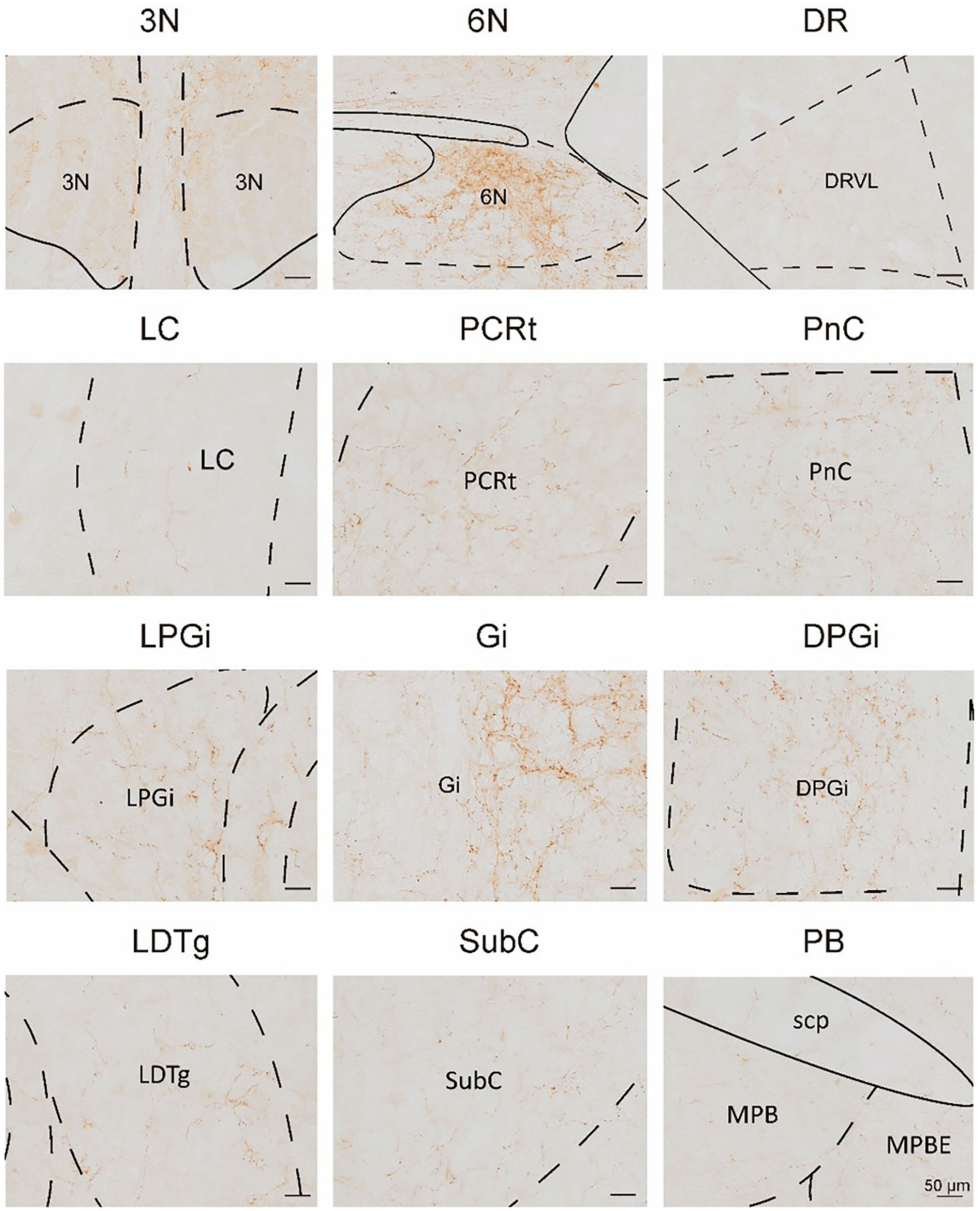
Multisensory functional nuclei that VN GABAergic neurons innervated. VN GABAergic neurons projected to nuclei that regulated eye movement (e.g., 3N and 6N), sleep-wake cycle (e.g., DR, LC, PnC, LPGi, Gi, DPGi, LDTg, SubC, and PB), and emotion control (e.g., LC and DR); scale bar: 50 μm.

In this study, we applied the cell-type-specific anterograde and retrograde tracing techniques dependent on a cre/loxp transgenic mice line to show comprehensive neural connectivity of VN GABAergic neurons in the whole brain. It revealed that the majority of dsRed-labeled afferent neurons of VN GABAergic neurons originated from the ipsilateral brainstem, especially the pons. In the meanwhile, the axonal terminals of VN GABAergic neurons also lie in the ipsilateral brain area and mainly established anatomical connectivity with the brainstem. Compared with previous studies that have revealed some innervating nuclei of VN using non-specific retrograde vectors, this study identified novel neural circuits among VN GABAergic neurons and other brain regions. To be specific, the VN also receives projections from the following nuclei, namely 12N, APT, CGPn, CnF, ECIC, InG, IPL, Li, LRt, M1, M2, MdD, MdV, Mo5, P7, Pa6, PDTg, PnV, RC, Ro, Rob, RPO, RtTg, RVL, S1BF, Su5, VLL, and VTA (Figure 11). Additionally, VN also send outputs to the following novel nuclei, including PCGS, Pa6, Ro, MdV, 12N, Cu, Li, Mo5, and Gr (Figure 12). On the other hand, diverse functional areas built neural projections with VN GABAergic neurons, including 3N and 6N that maintain balance, some brainstem reticular regions, Mo5, and SubC that controlled the sleep-wake cycle, PAG and PB that mediated pain and the sleep-wake cycle, as well as LC and DR that regulated both emotion and sleep-wake cycle (Figures 13, 14).

### Neural circuity between VN GABAergic neurons and balance-related nuclei

GABAergic neurons in the VN, an essential part of the vestibular commissure system, sent nerve fibers to innervate nuclei that control the extraocular muscle and participated in the vestibulo-ocular reflex pathway. Additionally, when the uniform linear motion was not enough to trigger the vestibulo-ocular reflex, the GABAergic commissure system induces a velocity storage mechanism to enhance the stimulation of the vestibulo-ocular reflex pathway to maintain balance and gaze stability (Olabi et al., 2009). In addition, previous studies have shown that after receiving signals from vestibular end organs in the inner ear, the VN could innervate the ipsilateral 3N and contralateral 6N, eliciting the ipsilateral medial rectus and contralateral lateral rectus contract to produce an eye movement in the opposite direction from head movement (Scudder and Fuchs, 1992). Based on results in this study, VN GABAergic neurons had reciprocal neural connectivity with the ipsilateral 3N and contralateral 6N, and sent moderate outputs to 6N and only a few to 3N, indicating that the neural pathway between VN GABAergic neurons and 6N primarily regulated extraocular muscle movement. Next, VN GABAergic neurons simultaneously received moderate afferents from the contralateral 3N and 6N, suggesting that the contralateral 3N and 6N innervated VN GABAergic neurons to mediate the vestibular reflexes. In addition, VN GABAergic neurons only received dense inputs from the fastigial cerebellar nucleus (Med) but sent few outputs to Med, while our previous study revealed that VN glutamatergic neurons had reciprocal neural connectivity with Med (Shi et al., 2021). Med mainly received the stimulation from the vestibular system and controlled the extensor muscles of the limbs to maintain balance (Thach, 1970; Imperato et al., 1984; Siebold et al., 1999). Therefore, this suggested that Med projected to GABAergic and glutamatergic neurons in the VN to participate in vestibulo-ocular reflexes, thereby controlling posture and voluntary movements, while Med only received inputs from VN glutamatergic neurons dominating extensor muscles in balance control.

### Neural circuity of VN GABAergic neurons in the vestibular-autonomic pathway

Clinically, nearly 50% of dizzy patients with vestibular dysfunction, including vestibular migraine, vestibular paroxysmia, and vestibular neuritis, were associated with emotional disorders, including anxiety and depression. Meanwhile, patients with psychiatric disorders were more likely to present with vestibule-related symptoms than counterparts without psychiatric diseases. Although vestibular dysfunction is well-established to be associated with psychological symptoms, the underlying neural circuit has been not determined. 5-hydroxytryptamine (5-HT) neurons in the dorsal raphe (DR) and noradrenergic (NE) neurons in the locus coeruleus (LC) receiving inputs from the CeA dominated the emotional behaviors. We found that both GABAergic neurons and glutamatergic neurons in the VN established reciprocal neural connectivity with LC and DR, suggesting that VN might interact with LC-NE and DR-5-HT system to modulate vestibular-anxiety comorbidity with the release of excitatory neurotransmitter (glutamate) and inhibitory neurotransmitter (GABA).

On the other hand, vestibular migraine (VM) was the most common neurologic cause of vertigo in all adults and has a predilection in females (Neuhauser et al., 2006; Formeister et al., 2018; Beh, 2019), which presents with recurrent vertigo, migraine symptoms, and susceptibility to motion sickness (Lempert et al., 2012; Murdin et al., 2015). Recent studies utilized functional Magnetic Resonance Imaging (fMRI) in combination with vestibular assessment questionnaires to propose the decrease of the gray matter volume (GMV) in the prefrontal cortex (PFC) responsible for the VM (Zhe et al., 2020). In the meanwhile, the connection of PFC to the periaqueductal gray (PAG) and the central nucleus of the amygdala (CeA) contributed to the pain processing (Ong et al., 2019), and the reciprocal connection between CeA and PB participated in the chronic pain regulation (Raver et al., 2020). Our findings showed that PAG and PB sent moderate outputs to VN GABAergic neurons but only did PB receive equal projections from VN GABAergic neurons, while VN glutamatergic neurons built a bidirectional connection with PAG and PB (Shi et al., 2021). Taken together, we suggested that GABAergic and glutamatergic neurons in the VN received ascending nociceptive signaling from PAG and PB, and sent feedback information to counteract nociceptive centers. In addition, the functional connectivity between PAG and nociceptive regions in female was stronger than that in male (Gecse et al., 2021), which indicate that the PAG-VN pathway might result in the female susceptibility to vestibular migraine.

Interestingly, the psychological disorders (depression and anxiety) associated with neuropathic pain were affected by the neuroelectrical activities of the LC-NE system that innervates PFC (Alba-Delgado et al., 2013; Hirschberg et al., 2017). In addition, the direct monosynaptic projection from DR-5-HT to CeA is also involved in the comorbid emotional symptoms of chronic pain (Zhou et al., 2019). Furthermore, the incidence of vestibular migraine was the highest in the prevalence of psychiatric comorbidity than that of any other vestibular disorder (Lahmann et al., 2015). Therefore, we assumed that VN GABAergic and glutamatergic neurons modulated vestibular dysfunction-emotional disorders-pain comorbidity *via* the dual projection with DR and LC.

### Neural circuity between VN GABAergic neurons and sleep-wake nuclei

Previous clinical studies demonstrated that the disturbed sleep–wake state was accompanied by vestibular dysfunction, such as vestibular disorders, motion sickness, and zero gravity (Mutlu et al., 2015; Pandi-Perumal and Gonfalone, 2016). Additionally, the rocking experiment showed the simulation of vestibular otolithic organs increased non-rapid eye movement (non-Rem, NREM) sleep stage N2 and promoted sleep in the mice (Kompotis et al., 2019). Although the neural circuits among the neurovestibular system and autonomic nuclei were implicated responsible for the homeostatic and circadian function, such as sleep–wake disorders and autonomic dysfunction (Fuller et al., 2002), its direct neurotransmitters involvement and the contribution of underlying anatomical connectivity remained to be experimentally verified. Our results showed that VN GABAergic neurons also had reciprocal neural connectivity with LC, DR, and LDTg targeted by orexin neurons that controlled the sleep–wake cycle (Luppi and Fort, 2019), suggesting that orexin neurons in the lateral hypothalamus might interact with LC, DR, and LDTg to influence the role of VN glutamatergic and GABAergic neurons in the sleep regulation. Furthermore, the interaction among VN GABAergic neurons and ventral medulla (vM) (including Gi and LPGi) contributed to the REM sleep period in the sleep–wake cycle. Compared with the whole-brain mapping atlas of the VN glutamatergic neurons only VN GABAergic neurons build a bidirectional connection with Mo5 responsible for the movement of the mandibular muscles (including the masseter) as the largest intracranial motor neuron (Fay and Norgren, 1997; Shi et al., 2021). The REM period was accompanied by relaxation, phasic activity, or jerking of non-respiratory muscles, especially of the cranial muscles (e.g., eye, tongue, face, and jaw muscles) (Wang et al., 2021). Additionally, Mo5 received inputs from glutamatergic neurons in the PCRt-PMnR, thereby driving the phasic movement of the masseter muscle during REM sleep (Anaclet et al., 2010). In this study, the correlation among VN GABAergic neurons and PCRt and PMnR indicated that the contribution of this neural circuit capable of controlling masseter muscle movement leads to REM sleep modulation. Additionally, vlPAG/DpMe contained the largest number of REM sleep neurons in the brainstem and GABAergic neurons involved in the transition from NREM to REM sleep (flip-flop circuit) (Luppi et al., 2006; Sapin et al., 2009). Our results showed that vlPAG and DpMe sent outputs to VN GABAergic neurons, while DpMe only received inputs from VN glutamatergic neurons. Furthermore, vM inhibits vlPAG/DpMe with the release of GABA to promote REM sleep (Grace and Horner, 2020). In summary, our results indicated that the simulation of the vestibular system might promote REM sleep transition and this effect required the projections from VN glutamatergic neurons to the GABAergic neurons in the vlPAG/DpMe. On the other hand, our finding that vlPAG/DpMe also projected to GABAergic neurons in the VN indicated an underlying feedback mechanism between VN GABAergic and vlPAG/DpMe neurons, which might account for the vestibular dysfunction on the disturbance of REM sleep.

### Underlying neural circuity of VN GABAergic neurons in body-fluid homeostasis

This study showed that VN GABAergic neurons could also receive monosynaptic afferents from the paraventricular nucleus in the hypothalamus (PaLM/PaMM). The paraventricular nucleus was composed of small-sized neurons, large-sized neurons, and long-distance neurons. Among them, large-sized neurons were the key component in the PaLM/PaMM, which mainly targeted the posterior pituitary to promote the secretion of arginine vasopressin (AVP) from the thick lobe of the pituitary, thereby adjusting the body-fluid balance in the hypothalamic-neuropituitary system (HNS) and involving in feeding behavior (Swanson and Sawchenko, 1983). Clinically, patients with endolymphatic hydrops (Ménière's disease and delayed endolymphatic hydrops) presented with vertigo, and for those who present with motion sickness, the level of blood AVP was elevated (Xu et al., 2015). Therefore, we suggested that the paraventricular nucleus in the hypothalamus might regulate the release of AVP involved in the activity of VN GABAergic neurons on the maintenance of body fluid balance.

However, there are limitations in this study to be mentioned and warrant further study. This study focused on the GABAergic neurons in the whole vestibular nuclei complex other than each sub-nucleus. In view of a developmental standpoint, the vestibular nuclei complex ranged from rhombomeres r1 to r9, and different subnucleus origin in separate rhombomere could innervate different regions performing a distinct function (Buttner-Ennever, 1992; Diaz et al., 2003; Diaz and Glover, 2021). Therefore, it would be more interesting to investigate projections of each vestibular subnucleus at rhombomeric levels in our future study. In summary, we demonstrated a whole-brain neural mapping of VN GABAergic neurons and analyzed statistically the distribution of inputs and outputs in each brain subregion. Interestingly, this study revealed several novel nuclei could build direct anatomical connectivity with VN GABAergic, which has never been reported in previous studies. More importantly, our findings also indicated VN GABAergic neurons might establish neural circuits with some functional nuclei in the whole brain, especially regulating balance maintenance, emotion control, pain processing, sleep and circadian rhythm regulation, and fluid balance. This study improves understanding of the neural connectivity of VN in the whole brain, which might provide the anatomical foundation for further study on the neuropathological mechanism of the co-morbidities with vestibular dysfunction.

## Data availability statement

The raw data supporting the conclusions of this article will be made available by the authors, without undue reservation.

## Ethics statement

All experimental protocols were approved by the Committee on the Ethics of Animal Experiments of Fudan University (Permit Number: 20140226-024).

## Author contributions

X-BS performed the experiment, analyzed data, and wrote the article. JW performed the experiment and reviewed the article. F-TL, Y-BZ, and W-MQ analyzed data. C-FD and Z-LH conceived the experiment and reviewed this manuscript. All authors contributed to the article and approved the submitted version.

## Funding

This study was supported by National Natural Science Foundation of China (82171142 and 81570917 to C-FD; 81800910 to JW; 82020108014 and 32070984 to Z-LH; 82071491 and 31871072 to W-MQ), National Major Project of China Science and Technology Innovation 2030 for Brain Science and Brain-Inspired Technology (2021ZD0203400 to Z-LH), National Key Research and Development Program of China (2020YFC2005300 to W-MQ), the Shanghai Science and Technology Innovation Action Plan Laboratory Animal Research Project (201409001800 to Z-LH), Program for Shanghai Outstanding Academic Leaders (to Z-LH), the Shanghai Municipal Science and Technology Major Project, and ZJLab (2018SHZDZX01 to Z-LH).

## Conflict of interest

The authors declare that the research was conducted in the absence of any commercial or financial relationships that could be construed as a potential conflict of interest.

## Publisher's note

All claims expressed in this article are solely those of the authors and do not necessarily represent those of their affiliated organizations, or those of the publisher, the editors and the reviewers. Any product that may be evaluated in this article, or claim that may be made by its manufacturer, is not guaranteed or endorsed by the publisher.

## Supplementary material

Supplementary Figure 1 Novel nuclei received projections from VN GABAergic neurons and schematic diagrams in the brain atlas; scale bar: 50 μm. PCGS, paracochlear glial substance; Pa6, paraabducens nucleus; Ro, nucleus of Roller; MdV, medullary reticular nucleus, ventral part; 12N, hypoglossal nucleus; Cu, cuneate nucleus; Li, the linear nucleus of the medulla; Mo5, motor trigeminal nucleus; Gr, gracile nucleus.

Supplementary Figure 2 Novel nucleisent monosynaptic inputs to VN GABAergic neuron VN GABAergic neurons and schematic diagrams in the brain atlas; scale bar: 50 μm. RtTg, reticulotegmental nucleus of the pons; PnV, pontine reticular nucleus, ventral part; Su5, supratrigeminal nucleus; Mo5, motor trigeminal nucleus; CGPn, central gray of the pons; Pa6, paraabducens nucleus; Li, linear nucleus of the medulla; LRt, lateral reticular nucleus; MdD, medullary reticular nucleus, dorsal part; MdV, medullary reticular nucleus, ventral part.

Supplementary Table 1 Distribution comparison between afferent and efferent neurons of VN GABAergic neurons.
