# Supplementary figures and images for: Whole-brain monosynaptic outputs and presynaptic inputs of GABAergic neurons in the vestibular nuclei complex of mice
Source: Front Neurosci. 2022 Aug 26;16:982596. doi: 10.3389/fnins.2022.982596 (PMC9459096; doi:10.3389/fnins.2022.982596)

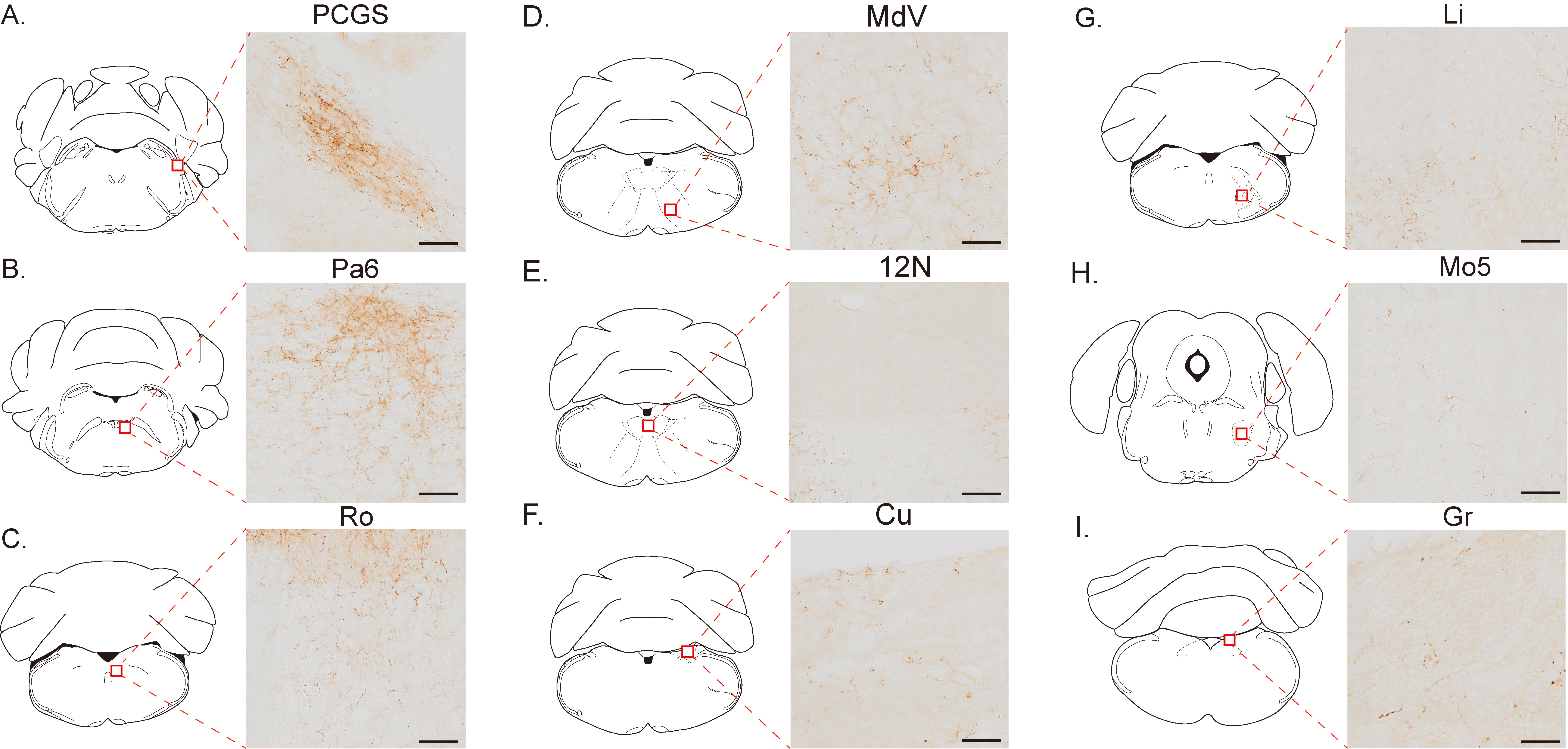

Supplement: Supplementary Figure 1 — Novel nuclei received projections from VN GABAergic neurons and schematic diagrams in the brain atlas; scale bar: 50 μm. PCGS, paracochlear glial substance; Pa6, paraabducens nucleus; Ro, nucleus of Roller; MdV, medullary reticular nucleus, ventral part; 12N, hypoglossal nucleus; Cu, cuneate nucleus; Li, the linear nucleus of the medulla; Mo5, motor trigeminal nucleus; Gr, gracile nucleus. [file Image_1.JPEG]

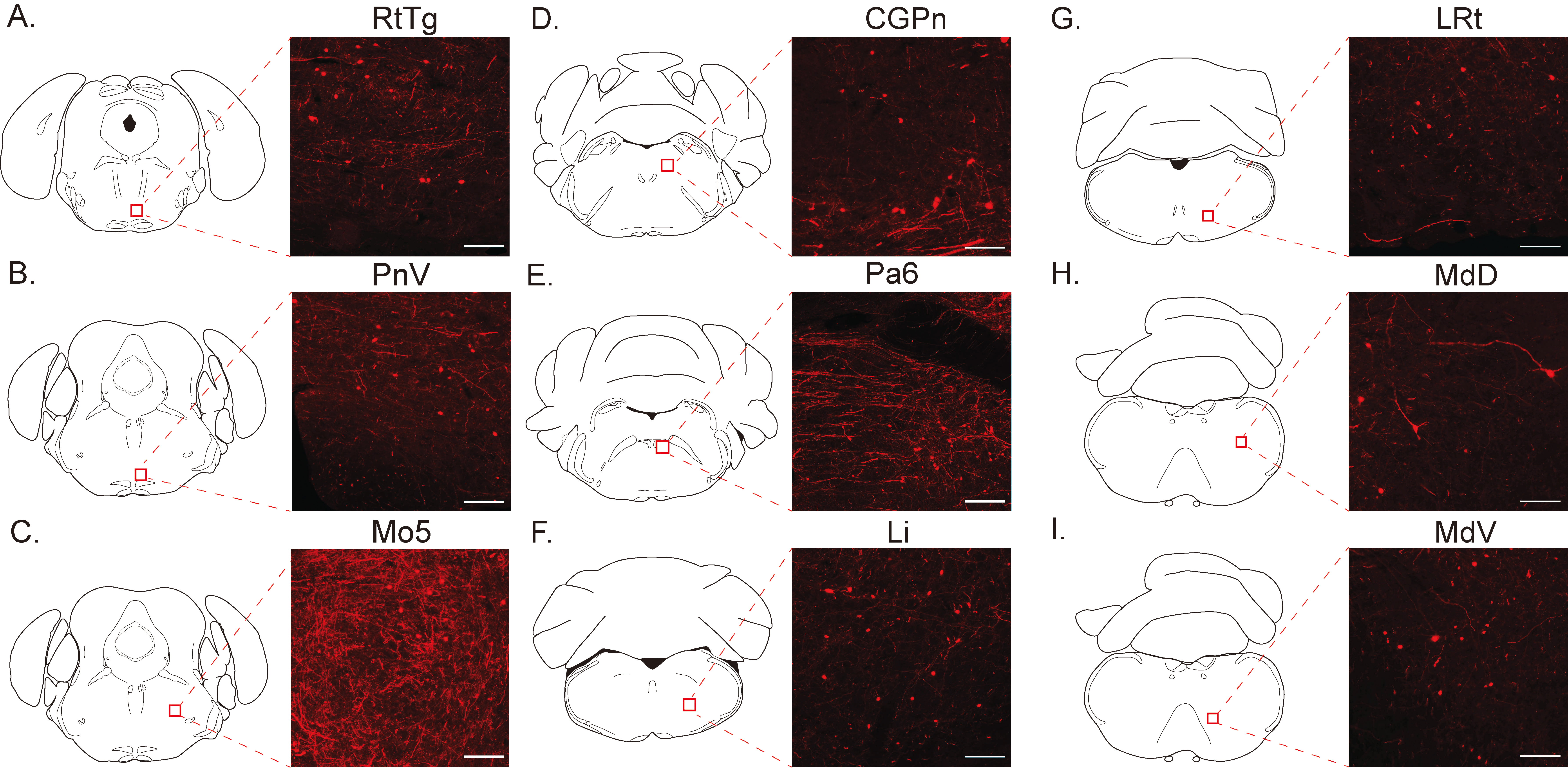

Supplement: Supplementary Figure 2 — Novel nucleisent monosynaptic inputs to VN GABAergic neuron VN GABAergic neurons and schematic diagrams in the brain atlas; scale bar: 50 μm. RtTg, reticulotegmental nucleus of the pons; PnV, pontine reticular nucleus, ventral part; Su5, supratrigeminal nucleus; Mo5, motor trigeminal nucleus; CGPn, central gray of the pons; Pa6, paraabducens nucleus; Li, linear nucleus of the medulla; LRt, lateral reticular nucleus; MdD, medullary reticular nucleus, dorsal part; MdV, medullary reticular nucleus, ventral part. [file Image_2.JPEG]
