# Supplementary material for: Whole-brain monosynaptic outputs and presynaptic inputs of GABAergic neurons in the vestibular nuclei complex of mice
Source: Front Neurosci. 2022 Aug 26;16:982596. doi: 10.3389/fnins.2022.982596 (PMC9459096; doi:10.3389/fnins.2022.982596)
Supplement: Supplementary Table 1 — Distribution comparison between afferent and efferent neurons of VN GABAergic neurons. [file Data_Sheet_1.PDF]

Table 1. Distribution comparison between afferent and efferent neurons of VN GABAergic neurons

Regions	Nuclei	Outputs	Inputs
Medulla	Pr	+++	+++
	DPGi	+++	+++
	PCRt	++	+++
	Gi	+++	+++
	IRt	++	++
	MdV	+++	++
	Sp5	+++	++
	Sol	++	++
	7N	++	++
	MdD	+++	++
	DpG	-	++
	LRt	++	++
	Li	++	++
	LPGi	++	++
	Cu	++	+
	Ro	++	+
	P7	+	+
	ECu	++	+
	12N	++	+
	RMg	++	+
	RVL	++	*
	Rob	+	+
	IO	+++	-
	10N	++	-
	Gr	+	-
Pons	PnC	+++	+++
	PnO	++	+++
	DMTg	++	+++
	SubC	++	+++
	Mo5	+	+++
	Pr5	+++	+++

	DpMe	-	+++
	Su5	-	++
	LDTg	++	++
	VLL	-	++
	PB	++	++
	CGPn	++	++
	KF	-	++
	Pa6	++	++
	RtTg	-	++
	PnV	+	++
	LC	+	++
	DTg	-	++
	MnR	-	++
	6N	++	++
	Bar	-	+
	PMnR	++	+
	RPO	+	+
	PDTg	-	+
	PCGS	+++	-
	SGI	++	-
	DC	++	-
	CPO	+	-
	CGA	+	-
	VPO	+	-
	MVPO	+	-
	LVPO	+	-
Midbrain	3N	+	++
	DR	+	++
	VTA	-	++
	InC	-	++
	Su3	+	++
	PAG	-	++
	3PC	+	++
	RC	-	++

	PPTg	-	++
	RPC	-	+
	CnF	-	+
	Dk	+	+
	IPL	-	+
	Su3C	-	+
	SN	-	+
	APT	++	+
Cerebellum	VeCb	+++	+++
	Med	-	+++
	IntP	+++	++
	Lat	-	+
	IntA	-	+
Inferior colliculus	InG	-	+
	ECIC	-	+
hypothalamus	ZI	-	++
	LH	-	+
	PH	-	+
	PR	-	+
	PaLM+PaMM	-	+
Cerebral cortex	M1	-	+
	M2	-	+
	S1BF	-	+
